# Real-time locating systems (RTLS) in healthcare: a condensed primer

**DOI:** 10.1186/1476-072X-11-25

**Published:** 2012-06-28

**Authors:** Maged N Kamel Boulos, Geoff Berry

**Affiliations:** 1 Faculty of Health, University of Plymouth, Drake Circus, Plymouth, Devon, PL4 8AA, UK; 2 International Society for Photogrammetry and Remote Sensing, Commission IV - Geodatabases and Digital Mapping, WG IV/4 - Virtual Globes and Context-Aware Visualisation/Analysis, ISPRS Headquarters (2008–2012), National Geomatics Centre of China, Beijing, 100048, People's Republic of China; 3 Real Time Location Technologies Ltd, Rotherham, S60 1FG, UK

**Keywords:** Real-time locating systems, Indoor tracking, Assets and individuals tracking, Healthcare optimisation

## Abstract

Real-time locating systems (RTLS, also known as real-time location systems) have become an important component of many existing ubiquitous location aware systems. While GPS (global positioning system) has been quite successful as an outdoor real-time locating solution, it fails to repeat this success indoors. A number of RTLS technologies have been used to solve indoor tracking problems. The ability to accurately track the location of assets and individuals indoors has many applications in healthcare. This paper provides a condensed primer of RTLS in healthcare, briefly covering the many options and technologies that are involved, as well as the various possible applications of RTLS in healthcare facilities and their potential benefits, including capital expenditure reduction and workflow and patient throughput improvements. The key to a successful RTLS deployment lies in picking the right RTLS option(s) and solution(s) for the application(s) or problem(s) at hand. Where this application-technology match has not been carefully thought of, any technology will be doomed to failure or to achieving less than optimal results.

## State-of-the-art review

**Figure 1:**
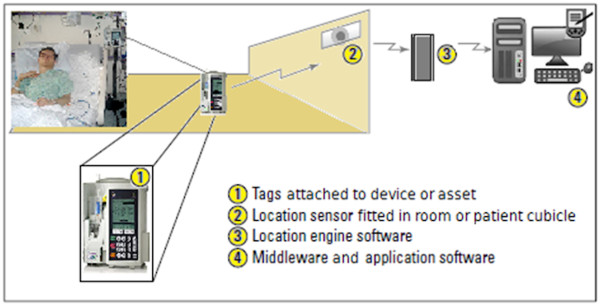
**Basic components of an RTLS (modified from**[1]**).**

Real-time locating systems (RTLS, also known as real-time *location* systems) are local systems for the identification and tracking of the location of assets and/or persons in real or near-real-time. An RTLS consists of specialised fixed receivers or readers (location sensors) receiving wireless signals from small ID badges or tags attached to objects of interest and/or persons, to determine where the tagged entities are located within a building or some other confined indoor or outdoor space (Figure 1). Each tag transmits its own unique ID. The tag ID is logged against the asset or person to which/whom it is attached. The tags periodically transmit their ID, and depending on the technology chosen, the system locates the tags (and therefore the tagged entities) within a few rooms on one of several floors or to a specific room or part of a room on a specific floor. When staff members require portable assets, they log onto the system at a workstation (or using a mobile device), identify where the closest available item is located, and go and get it.

RTLS location information typically does not include complete or continuous navigation details such as speed, direction, or spatial orientation of tracked assets and persons. Standards governing RTLS include ISO/IEC 24730 standards series, which describes a form of RTLS used by a subset of vendors, but does not cover the full range of RTLS technologies [2].

Emergency first response, healthcare and hospitals [3-6], care homes [7] and even everyday home life (as an assistive technology, where applicable) can all potentially benefit by using an appropriate RTLS solution.

## RTLS components and technologies

In an RTLS, the location engine software communicates with tags and location sensors to determine the location of tagged entities. The location engine relays this information to specialised middleware and applications. The middleware in an RTLS acts as the “plumbing” between the core RTLS components (tags, location sensors and location engine software) and a range of software applications capable of displaying and exploiting the real-time location and status information of tracked entities [1]. These latter applications vary from simple RTLS end-user interfaces for querying and displaying location information of tagged entities to more comprehensive integration into (or interoperability with) existing business/enterprise systems such as hospital ADT (admission, discharge and transfer) systems and HIS (hospital information systems and subsystems such as RIS (radiology information systems), operating room (OR) systems, bed management systems, etc.) via standards-based, open APIs (application programming interfaces) to enrich these systems with location information necessary for the completion of a variety tasks and process flow management operations [4,8].

The location engine, middleware and application software may run on the same computer or on different machines. These applications also often offer some kind of client interface such as a Web-browser-based or mobile interface [1].

Tags can also be equipped with push buttons (or call buttons). These can be used as panic buttons for summoning emergency response. Whenever the person carrying the tag presses the panic button, the location engine raises an alert and provides the location of the individual who pressed the button. Another use of tags with push buttons is when they are attached to assets, staff can use the button to indicate or toggle asset status, such as ‘bed occupied by patient’ (hospital bed status) or ‘device in need of repair’, as appropriate [1].

If a tag has voice-to-voice capability, it can be used to communicate with the individual carrying the tag based on his/her location. Buzzers (emitting sounds, recorded voice messages, or live messages), LEDs (light emitting diodes of different colours and blinking patterns), or LCD (liquid crystal display) screens (displaying text messages) can also be fitted on tags to communicate information or alerts to the person carrying the tag, to identify or locate an asset, and to communicate with the person who has the asset or is expected to check the asset [1].

Location sensors too can be fitted with buzzers. Patient ID badges sometimes become buried in bed linen (when patients are discharged) and cannot survive the bed linen wash cycle. In this case, installing location sensors that can sound an alarm whenever badges are detected in laundry chutes could prove helpful [6].

Various sensors can be incorporated in tags to gain information about the environment, the status of the person carrying the tag, or the tagged asset. For example, motion sensors in the tag can indicate whether the individual carrying it is moving, while a temperature sensor attached to a device can indicate whether that device is in optimum thermal operating conditions [1].

Tags can also have connectors that connect to assets in order to communicate specific details about the asset or its operational state. For example, the tag can indicate not only where the tagged device is, but also whether it is powered on. Finally, tags can have writeable memory, e.g., to log and store some user and other data about the tagged asset [1].

When tracking the physical location of an asset or an individual, depending on the needs of the application(s) at hand, we may want to know the absolute position (absolute coordinates, such as latitude, longitude and altitude), relative position (distance in three dimensions with reference to a fixed point, e.g., the nurse is standing at 10 metres north of the main entrance of the ward), or symbolic position (presence in a specific area, e.g., the surgeon is in operating theatre A, or presence near something or someone, e.g., the nurse is near patient B) [1].

To meet the requirements of different applications, whether they need precise location or room-level location, various RTLS solutions are available that can report tag location at different resolutions [1]:

*Presence-based locating*: RTLS returns tag location as to whether it is present in a given (relatively wide) area;

*Locating at room level*: RTLS returns tag location as present in a specific room, e.g., if a nurse presses the panic button to summon security assistance in the event of a physical attack on her, the location engine reports the nurse’s exact room in the hospital to the security personnel;

*Locating at sub-room level*: RTLS locates tag to a specific part of the room, e.g., in hospital rooms accommodating multiple patients, such as dual-bed rooms and larger wards, if a nurse is carrying a tag, the location engine can report how much time the nurse has spent by each patient’s bedside or cubicle;

*Locating at choke points*: tag location is returned by a specific choke point (an entry or exit point, such as a ward entrance; it is assumed that individuals or assets move from one area to another through these points). By monitoring the time a tag was detected at specific points, one can also determine the direction the tag is moving;

*Locating by associating*: tag location is returned as proximity with respect to another tag, e.g., if each patient in a hospital wears a tag and each IVF (intravenous fluid) pump has a tag, the location of a given IVF pump can be returned as present next to a specific patient (and for how long); and

*Locating precisely*: the exact tag location is pinpointed precisely on a map of the world and/or a detailed indoor map/in a given building and reported as absolute or relative position as described above.

It is worth noting here that business cases for true real-time systems are very rare. For most common RTLS applications, the requirement is to know where someone or something is located, when he/she or it is required. In this respect, the system needs to provide real-time location information simply when the information is needed and not continuously; it does not need to update the information every few milliseconds. In terms of cost, it may be cost effective to rationalise this issue when scoping a project. The question is why identify where assets are located each second when they only move once every 1–2 hours or days, in which case identifying where they are each minute would be more than adequate; the technology would also be less stressed and system costs are likely to be reduced.

An RTLS can be realised using various technologies, including light, camera vision, infrared (IR), sound, ultrasound, Bluetooth, Wi-Fi, RFID (radio frequency identification; RFID tags can be either active, with a small power supply to send out a signal covering a range of up to 100 metres, or passive, with no power supply and activated by a scanning signal, which limits their range of detection to less than a metre), ZigBee [9], ultra-wideband (UWB), GPS (global positioning system) and Cellular, among other technologies [1,10,11]. Different technologies use different approaches, and each method supports different applications or solves a slightly different problem while introducing its own limitations, e.g., IR requires a clear line of sight for the tags and sensors to communicate, so if a badge worn by a patient is covered by a blanket or flipped around, the system might momentarily lose track of the patient [6].

These technologies also vary in many aspects, such as the physical phenomena used for location detection, the tag’s form factor and that of the associated location sensors, power requirements/battery life, range, indoor versus outdoor applicability, installation and maintenance/scalability considerations (which also affect cost—[3,8]), and cost vs. time and space resolution (or precision; for example, the physics of Wi-Fi radio frequency, which passes through walls, limit accuracy to floor level at best and certainly not room-level precision [3], but one should note here that not all applications will require the same or the highest levels of accuracy). Some technologies require additional location sensors, and some leverage existing infrastructures, such as electricity or Wi-Fi in the building [1,3,11]. Some tag properties might also be essential for certain applications, e.g., waterproofness/water resistance and whether tags could be autoclaved [8].

In some systems, the tag being located actually computes its own position (tag self-positioning), while in other RTLS, the software that locates the tag is external to the tag (remote-positioning), or tag position is determined by recognising the location of a nearby tag (tag indirect-positioning) [1].

In the end, all RTLS technologies share the common objective of determining the location of assets and individuals as precisely as is needed by the target application(s). Each technology will succeed in its own way, provided it has been carefully matched to suitable applications. However, where this application-technology match has not been carefully thought of, any technology will be doomed to failure or to achieving less than optimal results [12]. For certain applications, the use of ‘best of breed’ or blended RTLS solutions that incorporate complementary technologies such as IR and RFID can deliver levels of precision and flexibility that are unachievable by any single competing technology [3].

## RTLS applications in healthcare

RTLS can be used to quickly locate healthcare staff in large facilities when a patient or other member of staff summons assistance during a medical emergency. RTLS can also be deployed to track the physical movement of patients to help ensure their safety, particularly in the case of Alzheimer’s and dementia patients. An RTLS can alert staff and pinpoint the location of a resident who wanders away from a pre-defined area or tries to leave the building, e.g., when a patient passes too close to an entrance or an exit. Automatic door locking may also be triggered in such cases, as appropriate [1]. (A related outdoor tracking application for Alzheimer’s patients using GPS is described in [13].)

Because locating by associating can provide detailed data on who is near whom, it can be used to detect how long a nurse has attended to a patient. A similar low-cost system for care homes can record each time care assistants attend to residents in their rooms [1,7]. Moreover, in older care homes, RTLS can provide information about residents’ mobility around the home (e.g., by computing the daily distance walked by each resident based on the distance between each sensor he/she passed by; the latter (distances between sensors) are stored in the computer system), which can be used as an indicator of residents’ overall well-being and in detecting problems, such as when an older resident has not left his/her room or visited a toilet within a pre-set period of time [7].

Tracking patient flows for throughput management can help diagnose bottlenecks and tailor (and monitor the implementation of) appropriate solutions for problems such as extended waiting times, overcrowding and boarding in outpatient clinics, emergency departments/rooms (ED/ER) and post-anaesthesia care units (PACUs); bumped and late surgeries; and the lack of available routine inpatient and intensive care unit (ICU) beds [1,5,14,15].

Monitoring patient flow or movement (handoffs) between departments, e.g., transfer from ED to radiology department, is accomplished by giving each patient a unique tag to always carry with him/her. The time spent by patients in each location is logged by an analytic application. By monitoring the time patients spend in various rooms and departments around the hospital, the hospital management can decide whether they need to allocate more staff or equipment at different departments and stages of the patient’s journey [1].

Moreover, an RTLS can directly decrease patient waiting and transfer times by reducing the time needed to find staff or to locate a wheelchair, for example, to transport the patient. Using an RTLS also allows quickly locating equipment that is due for maintenance, testing or inspection, as well as a closer synchronisation of housekeeping (bed cleaning) with patient discharge, enabling faster bed turnaround rates as part of a hospital bed management system. The latter can track real-time notifications of patient or bed status (such as occupied, available, assigned, discharge ordered, cleaning, or not in service, etc.), enabling faster transport of patients and faster housekeeping [1,16].

Laskowski-Jones [6] and Whalen in [15] report impressive RTLS-enabled workflow efficiencies, including quantifiable significant cuttings in ED wait times, length of stay (LOS) and ‘left without being seen’ (LWBS) rates (actual figures for wait times, LOS and LWBS rate reductions can be found in [6,15]). The value of the intelligence gleaned from RTLS patient flow data can be maximised by combining it with ‘lean production system principles’ (pioneered by Toyota Motor Corporation) to optimise patient flows [6,17-19]. Other benefits of patient flow tracking and optimisation include fewer ambulance diversions and higher patient satisfaction ratings [5], which can translate into improving the care facility’s perception and reputation.

Tracking expensive or shared equipment, such as ICU ventilators and intravenous (IV) pumps [20], can save time and money, and reduce equipment theft and accidental loss [1]. Hospitals are often large institutions, and personnel often find it difficult to locate portable equipment when it is required (Table 1 lists some examples of portable hospital equipment). Because personnel find it difficult to locate portable equipment when they need it, they sometimes “hide” (or “hoard”) it, so that they may find it when required; this practice exacerbates the problem.

**Table 1:** Examples of acute care hospital mobile assets

**Critical Care**	
· Adult/Paediatric Volumetric Pumps	· Foot Pumps
· Alternating Pressure/Flotation Devices	· Heat Therapy Units
· Ambulatory Infusion Pumps	· Hyper-Hypothermia Units
· Anaesthesia Machines	· IV Poles
· Bariatric Products	· Lymphedema Pumps
· Beds (specialty)/Rail Guards	· Patient Controlled Analgesia Pumps
· Blood/Fluid Warmers	· Sequential Compression Devices
· Cold Therapy Units	· Suction Devices
· Continuous Passive Motion Device	· Syringe Pumps
· Controllers, Infusion	· Tympanic Thermometry
· Defibrillators	· Ultrasonic Nebulizers
· Electrosurgical Generators	· Wheelchairs
· Enteral Infusion Pumps	
**Monitoring**	
· Anaesthetic Agent Monitors	· Telemetry Monitors
· Apnoea Monitors	· Urine Output/Temperature Monitors
· Blood Pressure Monitors	· Vital Signs Monitors
· Electrocardiographs	· Telemetry Monitoring Systems
· End Tidal CO_2_ Monitors	− Cardiac Care Systems
· Foetal Monitors	− Intensive Care Systems
· Neonatal Monitors	− PACU Systems
· Oximeters	− NICU (Neonatal Intensive Care Unit) Systems
· PO_2_/CO_2_ Monitors	− ER Systems
· Recorders and Printers	− OR Systems
· Surgical Monitors	
**Respiratory Therapy**	
· Aerosol Tents	· Nebulizers
· Air Compressors	· Oximeters
· BiPAP (Bilevel Positive Airway Pressure	· Oxygen Concentrators
· Cough Stimulators	· Suction Devices
· End Tidal CO_2_	· Simple Spirometry
· Heated Humidifiers	· Ventilators
**Newborn Care**	
·Blood Pressure Monitors	· Infant Warmers
· Breast Pumps	· Infusion Pumps
· Foetal Monitors	· Neonatal Monitors
· Incubators	· Oximeters
· Infant Ventilators	· Phototherapy Devices

Estimates indicate that hospitals will purchase 10% to 20% more portable equipment than actually required for operational needs, so that staff may find it when needed. Let us assume the example of a hospital originally planning to procure 600 IV pumps at GBP £3,250.00 each (total: GBP £1,950,000.00). With the deployment of a suitable RTLS, these figures can be reduced to 530 IV pumps for a total cost of GBP £1,722,500.00. This is a saving of GBP £227,500.00. Now, if the investment in the RTLS has cost GBP £97,000.00, the final savings after investment will be GBP £130,500.00, a 134.5% ROI (return on investment) with immediate payback time. For more expensive equipment such as ICU ventilators, the ROI can be much greater, even when assuming a 50% depreciation value of purchased equipment (which cuts RTLS savings to half).

Lower capital expenditure will also result in a reduction in the cost of depreciation (where applicable), and fewer assets (530 instead of 600 IV pumps in the above example) will translate into a proportionate reduction in storage and maintenance needs and costs. Furthermore, with an RTLS, medical personnel spend less time looking for equipment, thus increasing efficiency and productivity, as well as staff (and patients’) satisfaction.

By deploying RTLS to locate IVF pumps, one can also track whether members of staff are complying with regulations regarding proper disinfection between uses by different patients. To quantify the benefits of deploying such an application, one can consider the industry average costs spent and negative effects on reputation in case a violation citation is received [1].

Compliance with hand hygiene protocol in hospitals can significantly minimise the risk of nosocomial infections. RTLS can be used as a low cost method for recording when members of staff use hand sanitation stations before and after they enter and leave rooms and wards. When a member of staff uses a hand washing station, a nearby electromagnetic field emitter (exciter) triggers the personal badge tag (active RFID) worn by the caregiver to transmit a ‘hand washing event’ message that identifies the caregiver and the time that the specific dispenser was used. The system is not used to micromanage individual members of staff, but is rather used as a hospital infection control measure to identify individuals and groups who may need additional training or education.

RTLS has the potential of improving the productivity of nurses and caregivers and hence their job satisfaction levels by reducing many mundane and repetitive tasks that staff encounters on a daily basis. For example, a nurse or a caregiver typically has to manually cancel a call (register that it has been answered), but an RTLS can perform the same task automatically by recognising the nurse’s presence in the room. RTLS can also cut the time staff has to spend to check the status of rooms and beds and also improve a patient’s family/visitors’ satisfaction by increasing their awareness of patient location [1].

An RTLS can be deployed as an important component of a comprehensive hospital security solution. Instances of physical and verbal abuses of nurses and other members of staff (by abusive patients, visitors and other staff) in healthcare facilities, especially psychiatric hospitals, are not uncommon. RTLS can improve the safety of staff and nurses by giving them a means to request emergency assistance during crisis situations. Moreover, tracking personnel also alleviates security concerns by monitoring unauthorised access in restricted areas [1]. However, RTLS can be perceived as ‘big brother’. It is therefore important to promote its operational benefits to stakeholders prior to implementation and include appropriate checks to ensure their privacy is not infringed.

In 2001, the second author was involved in an RTLS deployment at a major London hospital which failed. The project was to install a nurse call/nurse tracking system within a new wing in the hospital. The scope of the project in the beginning was to improve safety procedures for nurses within the hospital. It had been noted that physical attacks and verbal abuse of nurses was occurring almost daily. In order to address this, hospital management decided to implement a system that enabled the nurses to raise an alarm and alert security personnel to nurse’s precise location when an incident occurred. The hardware was installed in the new wing and the nurses were issued each with an ID badge; the badges were fitted with a distress button and transmitted the ID and therefore the nurses’ locations as they moved around the wards. In addition to the nurse tracking system, a nurse call system was also required for the new wards, and it was decided that the nurse call system and the RTLS systems should be integrated. This function would allow management to identify where nurses were located when a nurse call event was activated, what type of event they were dealing with at the time, and how long they took to respond to nurse calls. But the nurses refused to comply with the system (they did not wear the ID badges) and therefore could not be tracked. As a result of this, the system was never used, hence the importance of educating users and addressing any privacy or other concerns they might have.

## Discussion, practical recommendations and conclusions

In healthcare facilities, RTLS can be used to locate portable assets and equipment, locate staff quickly and efficiently, and improve workflow. Hospital throughput of patients can be improved by ensuring the correct medical staff and equipment are in the correct place at the right time.

It is important to keep in mind that when vendors are more knowledgeable than the people procuring anything complex, the potential for dissatisfaction is likely to be present. Healthcare procurement teams should not simply take vendors’ marketing information and glossy brochures at face value. Moreover, the impact of the lack financial stability of many of the RTLS players within the industry in today’s (2012) gloomy global economic climate (particularly among the smaller vendors/system integrators) means that vendors are often desperate for revenue; under the circumstances, they may be compelled to offer and sell their products or “solutions” for any healthcare-related project, even when they do not have the correct solution for the client. This could be summarised as ‘*to a hammer, every problem looks like a nail*’.

Many of the companies providing products are several years from profitability, they are investing in building a sales and customer support infrastructure, and it is unlikely that all of them will survive to maturity. The problem for the clients of such companies will be how to support legacy (closed, proprietary) systems if the vendors are no longer trading. To reduce such risks, clients should insist on procuring standards-based technologies that support open APIs [8].

Prospective clients should also ask vendors bidding for an RTLS installation to provide references from existing customers covering previously delivered work, particularly work of similar nature and requirements as the current job. They should then carefully check all references received (the second author knows of at least one company who has no installations, but nevertheless advertises several “references”).

Many IT (information technology) projects fail, particularly large ones; they are either abandoned prior to implementation (due to cost overruns), or they do not achieve the required functional or business benefits. There have been several well documented IT project failures (either partial or complete) in the healthcare sector, including the well-known case of the NHS (National Health Service) National Programme for IT in England. RTLS installations bring in additional factors that may lead to project failure.

The IT sector has an inbuilt expertise in protecting themselves from the consequences of project failure. Indeed, the techniques are taught in many universities; they are called ‘functional specifications’. The deliverables and system functionality of a proposed system are detailed in its functional specifications. This is the case for all major IT deployments, including RTLS installations. The outcome is defined in such a way that allows the project to be declared a success if it can be shown to have met its functional specifications, regardless of whether or not it has also met the requirements as sold and anticipated by the client. One problem here is that vendors are experts in the sciences of the hardware and marketing; they hard promote their hardware because they consider that to be their differentiator in the marketplace. Clients, on the other hand, are usually purchasing *a solution to an operational problem* (rather than a mere hardware installation), and misunderstandings can arise in the (fine, but often critical) details.

RTLS systems are high involvement products, and typically the evaluation, selection and procurement team will consist of a of a stakeholder panel drawn from only the procuring organisation. The panel will examine and evaluate the offers received in detail; often they quickly adopt the domain and terminology of the vendors. Vendors usually provide information about radio type and frequency of transmission, received signal strength (RSSI), triangulation, multiple paths, etc. Depending on the makeup of the selection panel, this may or may not be relevant information, because they may not be cognisant of the differences in the capabilities of products offered, due to minutiae. Unfortunately, although the product details supplied by the vendors may be accurate, buyers responsible for the procurement of RTLS for the first time may not be aware of the consequences of decisions based on minutiae provided by vendors.

The choice of RTLS technology must be very carefully made. A given technology or hardware may not work well despite all its merits, if not properly matched to the intended application or the care facility’s (physical) environment, budget and future expansion plans (the latter will require an adequately scalable RTLS solution). For example, radio signals are susceptible to interference via signal propagation, metals, water, people, and radio signal collisions. Not every environment is suited for RF (radio frequency) systems.

Procurement teams should ideally include as many stakeholder groups as is possible in the whole process, from its beginning till final delivery. This is also needed to avoid cultural and organisational resistance to new procedures and working practices introduced by a new system and to successfully manage the associated organisational change and stakeholders’ adaptation to the new workflows. It should not just be assumed that everyone will willingly agree to all changes, because they seem like a good idea to senior management.

It is also advised (whenever possible) to invite a member of the vendor’s team to serve on the project panel from an early stage (once a suitable vendor has been picked). This enables the vendor to give advice on on-going system changes and enhancements at the ideas stage, rather than the vendor being presented with evolving requirements that are proposed by the client and then either attempting to “shoe horn” them into the system or negotiate changes after the fact.

Healthcare institutions should aim at improvements which are well within the capabilities of the technology and require modest procedural changes on behalf of users. They should make incremental changes and keep them simple [8].

Finally, selection and procurement teams should focus on achievable and demonstrable real-world benefits such as cost savings, improved efficiency, improved staff and patients’ satisfaction, etc. rather than on mere system specifications, making sure that any chosen vendor is committed to achieving these benefits. Bandi [8] also suggests partnership with vendors in a shared risk acquisition model. Vendor selection should always include a ‘Plan B’: what happens if the vendor fails; is there a contingency to source replacement hardware and obtain software support in this event?

## Competing interests

MNKB has no competing interests. GB is the founder and CEO (Chief Executive Officer) of a private company offering RTLS solutions.

## Authors’ contributions

MNKB conducted the literature review, identified and reflected on the main trends in the field, and conceived and drafted the manuscript. GB contributed expert vendor insight to the paper. Both authors read and approved the final manuscript.
